# Dynamics of fingertip contact during the onset of tangential slip

**DOI:** 10.1098/rsif.2014.0698

**Published:** 2014-11-06

**Authors:** Benoit Delhaye, Philippe Lefèvre, Jean-Louis Thonnard

**Affiliations:** 1 Institute of Neuroscience (IoNS), Université catholique de Louvain, Brussels, Belgium; 2 Cliniques Universitaires Saint-Luc, Physical and Rehabilitation Medicine Department, Université catholique de Louvain, Brussels, Belgium; 3 Institute of Information and Communication Technologies, Electronics and Applied Mathematics (ICTEAM), Université catholique de Louvain, Louvain-la-Neuve, Belgium

**Keywords:** skin mechanics, friction, touch, tactile perception

## Abstract

Through highly precise perceptual and sensorimotor activities, the human tactile system continuously acquires information about the environment. Mechanical interactions between the skin at the point of contact and a touched surface serve as the source of this tactile information. Using a dedicated custom robotic platform, we imaged skin deformation at the contact area between the finger and a flat surface during the onset of tangential sliding movements in four different directions (proximal, distal, radial and ulnar) and with varying normal force and tangential speeds. This simple tactile event evidenced complex mechanics. We observed a reduction of the contact area while increasing the tangential force and proposed to explain this phenomenon by nonlinear stiffening of the skin. The deformation's shape and amplitude were highly dependent on stimulation direction. We conclude that the complex, but highly patterned and reproducible, deformations measured in this study are a potential source of information for the central nervous system and that further mechanical measurement are needed to better understand tactile perceptual and motor performances.

## 1. Introduction

During object manipulation or tactile exploration, humans experience frequent partial or complete relative slippages between their fingertips and a contact surface. These events provide information about the mechanical properties of the surface (e.g. friction, surface roughness, shape, etc.). Previously, we showed that complete slippage occurs gradually, with the first ‘incipient’ slips occurring at the periphery of the contact, and an annulus of slip forming around a remaining ‘stuck’ zone [1]. As the tangential stress increases, this slipping area grows from the periphery to the centre, until the whole contact slips. This ‘stick-to-slip’ behaviour has crucial implications in dexterous manipulation and haptics. At the early phase of an object's lifting, partial slips at the periphery of the contact can be readily measured. Considering the importance of cutaneous feedback in object manipulation [2,3], researchers have long thought that these partial slips were responsible for triggering a reactive grip force [4]. Thousands of mechanoreceptors are present in the fingerpad [5], which respond vigorously to various constraints [6]. Thus, each deformation event at the contact interface generates potential information for the central nervous system.

The stick-to-slip transition was first experimentally observed by optical techniques [7,8]. Later, André *et al*. [1] performed a more in-depth analysis of the ‘stick ratio’, defined as the ratio of the stuck area to the contact area of the fingertip. They found a negative linear relationship between the stick ratio and increasing tangential force, with a slope that is inversely correlated to the normal force. In addition, they found a major influence of finger moisture on the stick-to-slip behaviour, with a moist finger reducing the tendency of a contact to slip.

This phenomenon finds theoretical grounds in contact mechanics. When two elastic bodies are normally loaded (called a ‘Hertzian contact’), the addition of a tangential force produces a theoretically infinite shear stress at the boundary of the contact, which results in peripheral partial slips. Mathematical equations describing the evolution of the stick ratio in the case of a Hertzian contact were obtained independently by Cattaneo [9] and Mindlin [10] (see Johnson [11] for a review). Using this mathematical framework to interpret the experimental data, Tada *et al*. [12] found only coarse qualitative agreement between model prediction and measurements of the evolution of the stick ratio. They also hypothesized that the indentation depth and sliding speed affect the propagation rate of the slip region.

In a recent survey, Adams *et al.* [13] considered a Hertzian pressure distribution [14–16] in the case of a light touch (0.5 N). For larger normal forces (5 N), they considered the approximation of a uniform pressure distribution in the contact area resulting in a linear relationship between the slip ratio and the tangential load. Consistent with the data of André *et al*. [1], they added an offset term in this relationship to account for the existence of a minimal tangential force required for the initiation of partial slips. Their linear model showed good first-order fit to some trials. Interestingly, the data of André *et al*. [1] also suggest a synchronous decrease in the contact area during the transition, although this observation was not quantified. This decrease presumably involves a ‘skin-peeling mechanism,’ in which some parts of the contact area lose contact during the transition.

Despite these studies, the stick-to-slip behaviour of the fingertips is not well understood and has not been quantified systematically. Therefore, we developed a robotic platform able to apply controlled stimuli to the fingertip, while the skin deformations are measured by optical means together with the contact forces. Using this system, we systematically explored different kinematics and dynamics of stimulation. The stimulation speed and forces were varied within ranges relevant to manipulation tasks and tactile exploration. As tactile stimuli can occur in any direction, our stimuli were applied in four different directions and we specifically focused on the effect of direction. Our analyses concentrated on the evolution of the contact area, and the localization and propagation rate of the slip region. We present our experimental data, together with a first-order explanation of their trend by modelling the contact as a Hertzian contact.

## 2. Material and methods

### 2.1. Data collection

#### 2.1.1. Subjects

Four healthy volunteers gave their written informed consent to participate in the experiment. The local ethics committee approved the study.

#### 2.1.2. Apparatus

**Figure 1.**
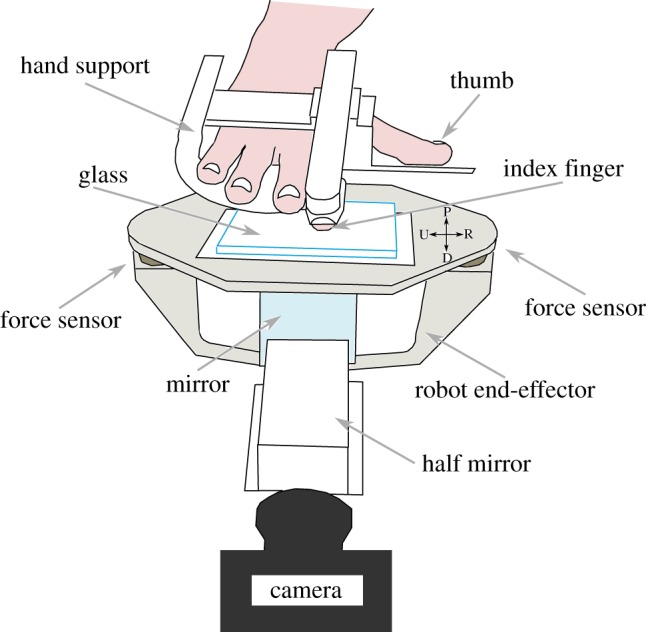
Apparatus used for the experiment. Top: Subject's hand is resting in the hand support, with the right index finger fixed. Middle: The end-effector of the robot (U-shaped, in grey) translates precisely in any direction. It bears the stimulus plate, made of smooth transparent glass, by two force/torque transducers. Bottom: Imaging system comprises a camera, a half mirror and a full mirror (detailed in figure 2). (Online version in colour.)

We developed a full custom robotic platform for applying controlled stimuli to the fingertip, as shown in figure 1. The platform is based on an industrial four-axis robot (DENSO HS-4535G) that can translate in three orthogonal directions. Its position is servo-controlled with a position resolution of 15 µm by a factory controller at a frequency of 1 kHz. The subject's index finger is fixed in a support that maintains a constant angle (around 20°) between the finger and the stimulus, which is a typical angle adopted during grasping and tactile exploration. With this small angle, the distal phalanx is nearly parallel to the surface [17]. The subject's nail is guided by a fixed piece of rigid plastic, which approximately has the same curvature as the nail and is hooked distally to block the nail position. The subject rests his or her hand and arm on the support.

The surface in contact with the index fingertip, called the stimulus, is a plate of transparent glass. The end-effector of the robot has a *U* shape. The stimulus is fixed to the end-effector with two force/torque transducers (ATI Nano43), which measure the normal and tangential forces that are applied to the fingertip along each direction (range: ±18 N, resolution: 0.004 N in each direction). A data acquisition board (NI PCI 6225) acquires the force signals at a frequency of 1 kHz. The normal force is fed back by a proportional-integral-derivative (PID) controller, which is tuned to keep the normal force constant at 0.5, 1 or 2 N.

**Figure 2.**
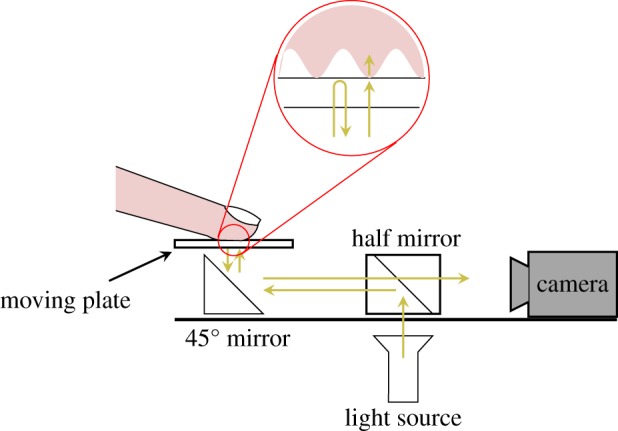
Imaging system. High-contrast fingerprint images are obtained by a coaxial light source and camera. (Online version in colour.)

The imaging system includes a camera that is fixed on the ground and not linked to the moving robot. The camera acquires images of the fingertip contact zone through the glass plate at high frequency (up to 200 fps) and high resolution (Mikrotron Eosens MC1362, 1280 × 1024 pixels, and around 1200 dpi). The fingerprint ridges are obtained at high contrast with a light reflection system [8] (figure 2). The camera has a coaxial light source, achieved by a half mirror. The light is either reflected or transmitted at the interface between the glass plate and the finger. Fingerprint ridges in contact with the glass plate cause the light to be scattered and transmitted into the finger, whereas the fingerprint valleys cause less light scattering. Thus, regions in contact with the glass plate appear much darker on the images. Images are acquired through a video acquisition board (NI PCIe-1433) mounted on a dedicated high-speed memory access computer. A TTL trigger is used to synchronize both acquisition boards. The camera is calibrated through the use of a reference frame that is painted on the glass surface and appears on the border of each frame.

#### 2.1.3. Experimental procedure

The following procedure was applied for each trial. (i) The robot end-effector was placed under the finger. (ii) The normal force controller was activated, and the glass plate was moved upwards to load the finger at a predetermined normal force. (iii) The camera recording was triggered, and the glass plate was moved 20 mm along a given direction at a constant speed, to generate a full slip of the index fingertip on the glass surface. (iv) The normal force controller was switched off, and the glass plate was moved down. This procedure was applied with three normal forces (0.5, 1 and 2 N) and three speeds (5, 10 and 20 mm s^−1^) along four directions of the glass plate's displacement relative to the fixed finger (distal—D, proximal—P, radial—R and ulnar—U). Each of the 36 conditions (3 forces × 3 speeds × 4 directions = 36 conditions) was repeated three times, for a total of 108 trials per subject in a randomized order (two blocks of 54 trials). The sudden increase in tangential force at the start of the movement produced a small error in the controlled normal force (less than 12%). Constant speed was reached in less than 150 ms.

### 2.2. Data analysis

Force signals were low-pass filtered with a fourth-order, zero-phase-lag Butterworth filter, which had a cut-off frequency of 40 Hz. The coefficient of dynamic friction (CDF) was evaluated as the ratio of the tangential force, *F*, to the normal force *W*, during the slipping phase, when the tangential force reached a plateau. The coefficient of static friction (CSF) was evaluated as the ratio of the tangential force to the normal force at the full slip onset (i.e. when the stick ratio reaches zero).

Images were sampled to obtain 10 equally spaced frames per millimetre of displacement (50 fps for 5 mm s^−1^, 100 fps for 10 mm s^−1^ and 200 fps for 20 mm s^−1^). As some trials showed poor image quality, the following criteria were applied for the selection of valid trials: (i) the detected contact area was larger than 20 mm^2^ and (ii) the contact area did not vary by more than 10% between two consecutive frames. According to these criteria, 380 of the 432 recorded trials were analysed.

#### 2.2.1. Apparent contact area

**Figure 3.**
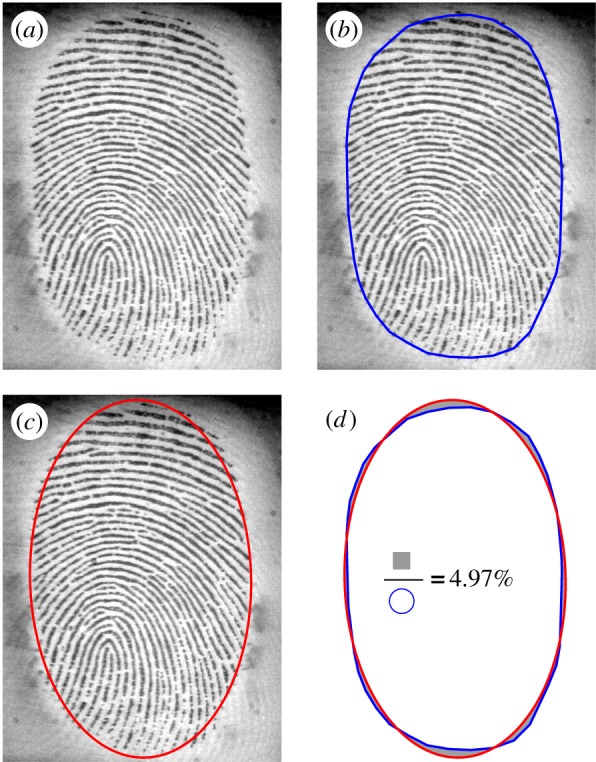
Contact area contour estimation. (*a*) Raw image of the fingerpad in contact with the glass. (*b*) Estimated contour in blue. (*c*) Fitted ellipse in red. (*d*) Error measurement between raw contour and ellipse fit (filled in grey).

The apparent contact area (referred to as the contact area below) contour of the finger on the glass surface was obtained by a three-stage process: (i) the images were band-pass filtered with homomorphic filtering [18]. The goal of this step was to correct for non-uniform illumination, and to retain only those spatial frequencies that were relevant to the fingerprint geometry (i.e. those with a periodicity around 0.4 mm). (ii) Grey-scale mathematical morphology (closing and then opening) was applied, to obtain a gross contact zone surrounding the fingerprints. The Otsu method was then used to provide a detection threshold for the border of the contact area [19]. The contact area value, *A*, (in mm²) was obtained by summing the number of pixel within the contact area and then scaling this value by the picture resolution (in pxl mm^−1^). (3) Fifty equally spaced point coordinates were sampled along the border of the contact area, and an ellipse was fitted on these coordinates by a least-squares algorithm [20]. The ellipse parameters (centre coordinates, long and short axes, and tilt angle) were computed and were used to compare position, aspect ratio and tilt angle of the contact zone before and during slipping. Figure 3 shows the result of contour estimation and ellipse fitted. An error index for the ellipse fitting was defined as the ratio of the error area (shaded in grey in figure 3*d*) and the raw contour area. The real contact area, *A*_real_, was obtained after segmentation in the contact area of the filtered images.

The contact area varies depending on the normal force, but data from André *et al*. [1] suggest that the contact area decreases during tangential loading. Any change in the contact area can be related to either a contacting or deformation mechanism. In what we defined a contacting mechanism, the change in contact area is due to some regions of the fingerpad coming into or losing contact with the surface, with contact loss being referred to as a ‘peeling mechanism’. In what we defined a deformation mechanism, the amount of tissue in contact does not change, but the change in the contact area is due to the compression or expansion of the tissue in contact with the glass. As the feature distribution in the contact area was nearly homogeneous (see §2.2.2. for feature sampling), the contribution of the contacting mechanism was roughly estimated by computing the ratio of the number of remaining features during slipping *N*_slip_ to the number of sampled features in the initial contact *N*_0_.

Hertz contact was used to interpret our results. We considered the case of a rigid flat surface (the plate of glass) loaded on a homogeneous and isotropic elastic sphere (the fingerpad). Hertz equation (2.1) gives the contact area, *A*, as a function of the normal force, *W*, the radius of curvature, *R*, and the reduced Young's modulus (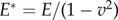, where *E* is Young's modulus and *ν* is Poisson's ratio [11]). In the case of the fingerpad, being a composite layered material with nonlinear viscoelastic and anisotropic response, the reduced Young's modulus is an effective value. Effective in the sense that a homogeneous elastic material would produce the same contact area as the fingerpad for the same *W* and *R*. As contact is elliptical, the radius of curvature is 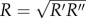 , with *R’* and *R’’* being the major and minor radii of curvature [21].2.1
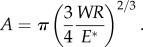


Even if the hypothesis of small contact relative to radius of curvature is violated, Hertz contact is a good first-order approximation [22,23].

The effective Young's modulus was measured at the initial contact by using equation (2.2) [24]. *∂δ* is the relative indentation from first instant of contact (*W* = 0) to stabilized contact force.2.2
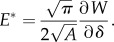


#### 2.2.2. Evolution of the stuck area

We used the optical flow technique to compute the stuck and slipping areas. The optical flow is a common technique used in computer vision to obtain a displacement vector field between two consecutive frames [25]. We implemented a custom C++ routine based on a standard open-source computer vision software library, which includes an optical flow implementation (OpenCV [26]).

The following procedure was applied to each image sequence. First, a maximal number of features were sampled over the whole contact area in the first frame. These features were selected according to optimal criteria in terms of tracking performances [27], and were nearly equally spaced (minimum spacing of 10 pixels, totalling around 2000 features). Second, features were tracked from frame to frame by the optical flow algorithm. Features with little similarity between consecutive frames or that crossed the border of the contact area were removed during tracking. The stimulus displacement, that is the plate of glass displacement relative to the fixed fingernail, was tracked from frame to frame, by using the previously described procedure on landmarks sampled on the reference frame (tracking RMS noise is below 0.2 pixels/frame, resolution 1200 dpi).

For each feature in the contact area, a relative displacement vector between the finger and the glass was obtained by subtracting the stimulus displacement from the feature displacement. A feature was considered to start to slip once a relative displacement of more than 50 µm was measured. The stuck area formed a single connected region and was well approximated by an ellipse. Therefore, an ellipse was fitted on this region following the same procedure as described for the contact area. We defined the stick ratio, *ϕ*, as the ratio of the stuck area to the contact area [1].

An error index was defined to quantify stuck area position and shape reproducibility. At a stick ratio of 0.5, the three stuck area contours (taken from the three repetitions) were compared pairwise. The same error measurement was done as in §2.2.1 (figure 3*d*) for each pair, leading to three error measurements. We took the median value of these three errors. This error measurement was done for each condition and each subject.

#### 2.2.3. Skin deformation margin

The first instant of full slip is defined as the moment when the stick ratio falls to zero. The displacement of the glass surface at this instant gives the precise displacement sustained by the skin just before complete slip, and we defined this displacement as the skin deformation margin. This measurement was made for every trial.

#### 2.2.4. Steady-state slip

Steady-state slip is defined as the state achieved during slippage when there is no relative displacement between tissues in the contact area. In steady-state slip, the relative speed between the finger and the glass is homogeneous in the contact area and equal to the glass speed, and the finger is stable relative to the camera. Steady-state slip occurs with some delay after the first instant of full slip, due to the presence of a speed gradient in the contact area at the first instant of full slip. We estimated the relative displacement between the instant of full slip and the instant of steady-state slip. The latter was obtained by computing the first instant when the speed of all features relative to the camera fell below a certain threshold (0.5 pixels per frame).

## 3. Results

**Figure 4.**
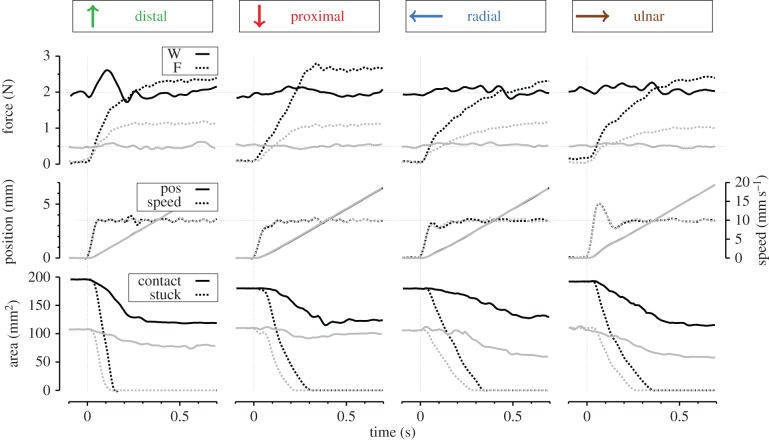
Individual trials in each direction from subject S3 at a speed condition of 10 mm s^−1^. Panels show the time evolutions of the contact forces (top), stimulus position and speed (middle), and finger contact area (bottom). Trials are aligned to the stimulus onset (thin vertical dashed lines). For each direction, two traces are presented: for a trial at high normal force (2 N, in black) and low normal force (0.5 N, in grey). In the top panel, solid lines represent normal force and dashed lines represent tangential force. In the middle panel, solid lines represent position of the stimulus and dashed lines represent speed. In the bottom panel, solid lines represent contact area and dashed lines represent stuck area. Stimulus directions (D, P, R and U) are relative to the fixed fingertip (figure 1). (Online version in colour.)

Figure 4 presents typical individual time-evolution traces of the contact force, stimulus position and speed, and contact/stuck area for each direction. A partial-slip phase can be defined between the onset of plate displacement and the first instant of full slip of the plate under the fingertip. During this phase, the contact area decreased, and the stuck area monotonically decreased to zero, which was defined as the instant of full slip. The duration of this phase—which depended on the direction of movement, the normal force and the speed—ranged from 90 to 980 ms across all trials. The contact area always stabilized to a constant value after the first instant of full slip.

Table 1 gives the mean value (and standard deviation) of the coefficient of dynamic friction, determined during the plateau of tangential force, for each subject and each level of normal force. CDF varied among subjects and trials. It decreased when the normal force increased (two-way ANOVA, *p* < 0.001), but was not influenced by the direction of plate displacement (*p* = 0.17). The value of the CSF, determined at *ϕ* = 0, was less than that of the CDF (mean difference: 0.31, paired *t*-test, *p* < 0.001) because the tangential force continued to increase slightly after the first instant of full slip.

**Table 1.** Parameters measured at the initial contact and during slipping for each subject and for the different loads.

subject	*W* (N)	CDF (–) mean ± s.d.	*A*_0_ (mm^2^) mean ± s.d.	*A*_slip_ (mm^2^) mean ± s.d.	E* (kPa) mean ± s.d.	*A*_0_ ∼ W^n^ *n*(*R*^2^)	*A*_o_ ∼ (*W*/E*)*^n^ n* (*R*^2^)
1	0.5	1.92 ± 0.39	83 ± 11	67 ± 13	30.4 ± 3.1	0.52 (0.84)	0.91 (0.93)
	1.0	1.60 ± 0.26	124 ± 12	90 ± 14	36.4 ± 2.7		
	2.0	1.29 ± 0.21	167 ± 15	112 ± 19	52.0 ± 4.1		
2	0.5	2.41 ± 0.33	92 ± 8	65 ± 9	35.9 ± 3.1	0.43 (0.89)	0.83 (0.91)
	1.0	1.92 ± 0.20	129 ± 10	82 ± 15	45.3 ± 3.9		
	2.0	1.53 ± 0.14	162 ± 3	101 ± 14	68.8 ± 5.3		
3	0.5	2.21 ± 0.31	116 ± 7	82 ± 12	27.7 ± 2.2	0.36 (0.87)	0.73 (0.87)
	1.0	1.73 ± 0.24	151 ± 14	97 ± 14	39.0 ± 2.6		
	2.0	1.33 ± 0.22	188 ± 5	119 ± 16	57.1 ± 4.5		
4	0.5	2.30 ± 0.42	97 ± 18	62 ± 12	30.8 ± 4.4	0.42 (0.62)	0.81 (0.78)
	1.0	1.88 ± 0.28	131 ± 23	81 ± 15	38.9 ± 4.8		
	2.0	1.54 ± 0.24	168 ± 22	102 ± 17	57.2 ± 6.9		

### 3.1. Apparent contact area

#### 3.1.1. Changes in the contact area

The mean values of the initial contact area, *A*_0_, and the steady-state slipping contact area, *A*_slip_, is shown in table 1 for each subject and each normal force. The contact areas were obtained by averaging values from multiple frames before the onset of the movement (six images) and during slipping (20 images). Variation across frames was low (standard deviation at initial contact: 1.9 ± 1.4 mm^2^ and during slipping contact: 1.2 ± 1.0 mm^2^, mean ± s.d. across all trials). Contact area differed between subjects. The contact area increased with normal force following a power law, with best-fit exponent around 0.4 as presented in table 1.

The effective Young's modulus measured for each subject is given in table 1. The contact area varied with the normal force *W* following a power law with best-fit exponent around 0.4. Moreover, the contact area also varied with the ratio *W*/E* following a power law, with best-fit exponent around 0.8 (table 1). The coefficients of determination (*R*^2^) of the later fit were higher.

**Figure 5.**
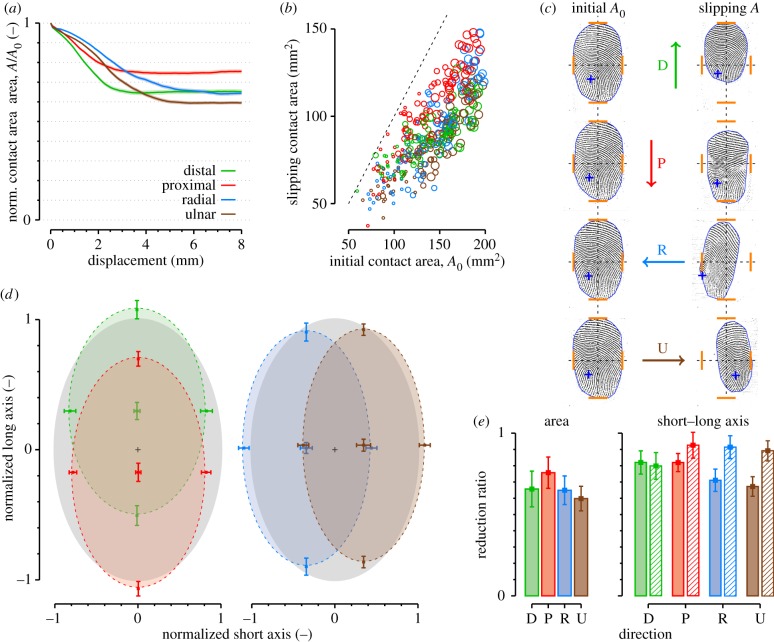
(*a*) Evolution of the contact area as a function of stimulus displacement. Solid lines are means among all trials. Shaded areas represent standard error. (*b*) Scatter plot of initial contact area versus slipping contact area during steady-state slipping. Circle sizes correspond to the three normal force conditions (0.5, 1 and 2 N). Direction of movement is represented by different colours. Dashed line represents equal contact areas (slope = 1). (*c*) Example images of the initial contact area (left) and slipping contact area (right) in each direction (D, P, R and U) from subject S3. Orange dashes report external limits (top, bottom, left and right) of the initial contact and a blue cross is attached to the papillary whorl. (*d*) Normalized shape of the contact region. Left: proximal and distal. Right: radial and ulnar. Grey ellipse represents normalized initial contact area. Coloured ellipses represent mean contact region during slipping. Central cross represents mean geometrical centre. Four corner points represent mean external borders of the contact area. Error bars represent standard deviation. (*e*) Reduction ratios between initial contact area and slipping contact area. Right panel: Shaded bars represent short axis, hatched bars represent long axis. Error bars represent the standard deviation.

Figure 5 describes the evolution of the contact area during tangential loading. Part (*a*) shows the evolution of the normalized contact area (i.e. relative to the initial contact area) as a function of plate displacement. The final area reduction and time course of reduction depended on the direction of movement (figure 5*a*,*e*, ‘Area’). The area reduction started earlier in the distal direction compared to the other directions. The mean reduction was relatively low for proximal movements (24%) and was similar in the other directions (around 35%) (figure 5*a* and *e*, ‘Area’). The contact area decreased systematically during the transition phase. For every normal force tested, the contact area was below the line of equality (dashed line in figure 5*b*).

Figure 5*c* displays one example for every direction (D, P, R and U) of a fingerprint image taken from subject S3. In this figure, the borders are highlighted in blue. On average, 93.2% of the total change in area was explained by a peeling rather than a deformation mechanism (*A*_slip_/*A*_0_ = 0.932 *N*_slip_/*N*_0_, on average, see §2.2.1.). This peeling mechanism occurred at different regions of the contact (figure 5*c*), depending on the direction of displacement. For the proximal and distal directions, the peeling was distributed uniformly along the periphery of the contact area. For the radial and ulnar directions, it mostly occurred on the side of the direction of motion (see in figure 5*c*, with blue crosses plotted on the papillary whorl as a reference).

The contact area could be very well approximated by an ellipse (error index, see §2.2.1, ranged from 2 to 15%). The direction of the stimulus affected the size of the contact area, as well as the shape and position of the final slipping area (figures 5*d*,*e*, ‘Short–Long axis'). In the distal direction, the short and long axes (of the ellipse that was fitted on the contact area) were equally reduced. In the proximal direction, the short axis was slightly more reduced than the long axis. In the radial and ulnar directions, the short axis was more reduced than the long axis (paired *t*-test, *p* < 0.001). Consequently, the contact area appeared more elongated in the radial and ulnar directions during full slip compared to the distal and proximal displacements (figure 5*d*,*e*, ‘Short–Long axis'). This observation is consistent with the directional effect of the peeling mechanisms described above.

#### 3.1.2. Modelling of contact area evolution

According to equation (2.1), under a controlled constant normal force, a change in the contact area can be attributed to a change in either *R* or E*. Here, we made the simplifying approximation that the change in the radius of curvature of the finger was small relative to that of E*. Therefore, the radius of curvature was considered a constant for a specific trial and E* as a variable, and changing as a function of the tangential force. That is, the skin gets stiffer when the tangential force increases. Without a good knowledge of the physical basis of such a change, we propose the simplest relationship between the two variables, a linear relationship (see equation (3.1)).3.1
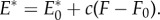


The subscript 0 refers to the instant when the tangential force is equal to zero (at the initial contact). We expected that an increase in the tangential force (*F*) would stretch the tissues tangentially such that they would become stiffer, with a slope of *c* (m^−2^). The offset term, *F*_0_, accounts for the existence of an initial increase in the tangential force that does not produce any change in the contact area (i.e. a tangential force for which the skin is behaving linearly). Using equations (2.1) and (2.2), the normalized contact area (i.e. ratio of the current to the initial contact area, *A*/*A*_0_) should change with the tangential force, as described by the following equation (with slope 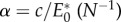).3.2
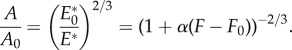


The two free parameters are the slope coefficient, *α*, and the offset term, *F*_0_. We used the MATLAB lsqcurvefit function to compute the best parameters to fit this simple model on every trial recorded.

**Figure 6.**
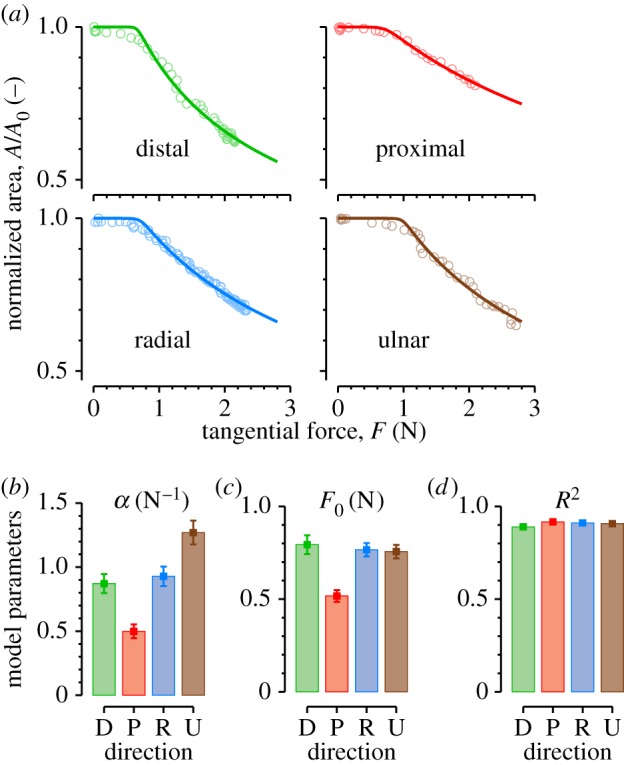
Evolution of the contact area with tangential force. (*a*) Typical traces for each direction from subject S2. Dots are data and lines represent best fit. Mean of best-fit parameters to equation (3.1), (*b*) *α*, (*c*) *F*_0_ and (*d*) coefficient of determination *R*^2^ pooled across all trials. Error bars correspond to standard error. Colours represent directions.

Typical trials from subject S2 and their fit are shown in figure 6*a*. The model is discontinuous at the transition between the linear and nonlinear part. The data show a more gradual transition between the two states. The means of best-fit parameters of equation (3.2) along each direction are shown in figure 6*b–d*. For each direction, there was a significant tangential force increase, *F*_0_, before the area began to decrease. This offset was smaller for the proximal direction (around 0.5 N) and higher for the other directions (around 0.75 N; see figure 6*c*). The slope, *α*, also varied depending on the direction. It was lower for the proximal direction (around 0.5) and higher in other directions (figure 6*b*). This parameter is strongly related to the final reduction ratio (figures 5*e* and 6*b* show the same trend). The *R*^2^ of the fits were close to one, showing a very good approximation of the data (*R*^2^ ∼ 0.9 in each direction, figure 6*d*). The averages of the area change, *A*/*A*_0_, along each direction ranged from 0.8 to 0.6 (see figure 5*e*, ‘Area’), corresponding to a theoretical Young's modulus change ranging from 1.4 to 2.15 (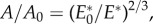
see equation (3.2)).

### 3.2. Stuck area

#### 3.2.1. Evolution with tangential force

**Figure 7.**
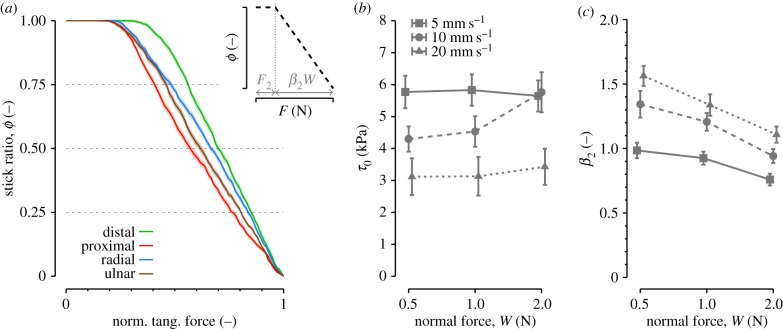
(*a*) Evolution of the stick ratio as a function of the normalized tangential force. Solid lines are means across all trials. Shaded areas represent standard error. (*b,c*) Variation in the estimated interfacial shear strength, *τ*_0_, (*b*) and slope parameter, *β*_2_, (*c*) as a function of the normal force and speed, all directions pooled. Error bars represent the standard error across trials.

Figure 7*a* shows the evolution of the stick ratio, *ϕ*, as a function of the tangential force, *F*. To present the pooled data from all trials, the tangential force was normalized by the slip force (i.e. the tangential force at the full slip onset). We found a linear decrease in the stick ratio as a function of the tangential force.

To characterize the evolution, two models from the literature were fitted to the data with the MATLAB lsqcurvefit function. The first model (M1, equation (3.3)) is the Cattaneo–Mindlin solution for partial slips during tangential loading in a Hertzian spherical contact [9–11]. An offset term was added to account for the possible existence of an initial increase in tangential force that does not produce any partial slip [13], resulting in equation (3.3). The two free parameters are the offset, *F*_1_, and the slope, *β*_1_.3.3
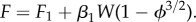


The second model (M2) is a linear model resulting from the assumption of a uniform pressure distribution in the contact area [13] (see equation (3.4)). The two free parameters are the offset, *F*_2_, and the slope, *β*_2_.3.4
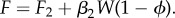


Linear model M2 (equation (3.4)) was the best candidate to fit the evolution of the stick ratio. Compared to model M1, model M2 gave a better coefficient of determination (*R*^2^) for every force and every direction tested (Bonferroni-corrected paired *t*-tests, all *p* < 0.05), except for conditions with a force of 0.5 N in the distal direction (*p* = 0.055) and the radial direction (*p* = 0.17).

The presence of a minimal tangential force to produce partial slip can be explained by the presence of an intrinsic interfacial shear strength, *τ*_0_ [28]. It was obtained from *F*_2_ = *τ*_0_*A*_real_ [13], *A*_real_ being the real initial contact area (*A*_real_/*A*_0_ average across subject was 0.57). Results are shown in figure 7*b* and values for *τ*_0_ are consistent with previous studies [13]. The minimal force was significantly higher for the distal than for the other directions (figure 7*a*). In addition, it increased with normal force and decreased with speed. The slope coefficient, *β*_2_, increased with speed and decreased with normal force (figure 7*c*), in agreement with the CDF.

#### 3.2.2. Shape and localization of the stuck area

**Figure 8.**
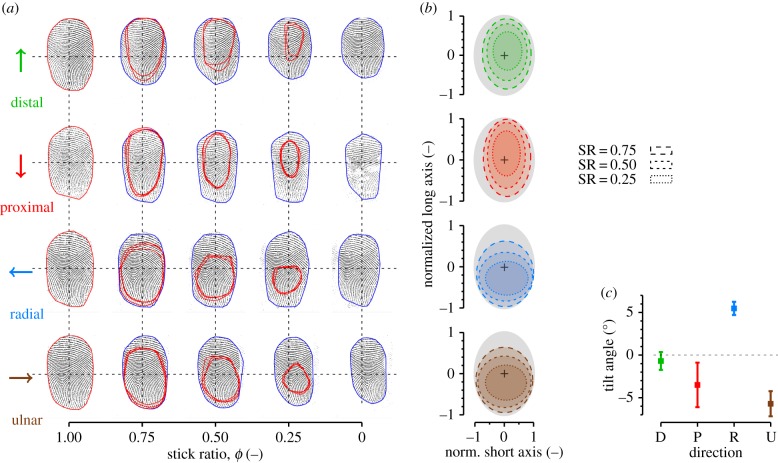
Evolution of the stuck area. (*a*) Example images of the evolution of the stuck area for stick ratios of 1, 0.75, 0.5, 0.25 and 0 for each direction for subject S3. Three trials (same normal force and same speed) are superimposed, with background picture of the contact area selected from one trial. Blue contours surround the contact area, red contours represent the stuck area. (*b*) Normalized mean shape of the stuck area pooled across all trials. Grey ellipses represent normalized contact region. Ellipses with dashed contour represent mean stuck area at different stick ratios. (*c*) Fitted ellipse tilt angles for each direction pooled across all trials. Error bars correspond to the standard error. (Online version in colour.)

Figure 8 shows the position and shape of the stuck area within the contact area. Part (a) displays typical images before the movement onset, during the transient phase, and after the full slip for each direction tested (D, P, R and U). Blue contours give the limits of the contact area, and red contours represent the stuck area. Three repetitions of the same condition and subject (S3) are overlaid (red curves), with a background fingerprint selected from one of the three repetitions showing a reproducible shape and location of the partial slips (error is 13.8 ± 5.2%, mean ± s.d. across all subjects and all conditions).

The orientation and position of the stuck area differed depending on the movement direction (figure 8*b*), and always tended to be longer in the direction of motion. In the proximal–distal direction, the main axis of the stuck area was aligned with the main axis of the contact area. The centre of the stuck area was slightly off-centred distally compared to the centre of the contact area (figure 8*b*, top). In these cases, the first micro-slips occurred everywhere at the periphery of the contact area and progressed to the centre of the contact area.

In contrast, the stuck area in the radial and ulnar movements was systematically off-centred proximally and did not keep the same aspect ratio (figure 8*b*, bottom). In these cases, most of the first micro-slips occurred at the distal periphery of the contact area, and progressed proximally later. Therefore, as the distal part slipped but the proximal part moved with the glass, the contact area tended to rotate systematically by about 5° for the radial and ulnar directions (*t*-test, *p* < 0.001; see figures 8*c* and 5*c*). No significant tilt was observed for the proximal and distal directions (*t*-test, *p* = 0.50 and *p* = 0.18, respectively).

### 3.3. Skin deformation margin

**Figure 9.**
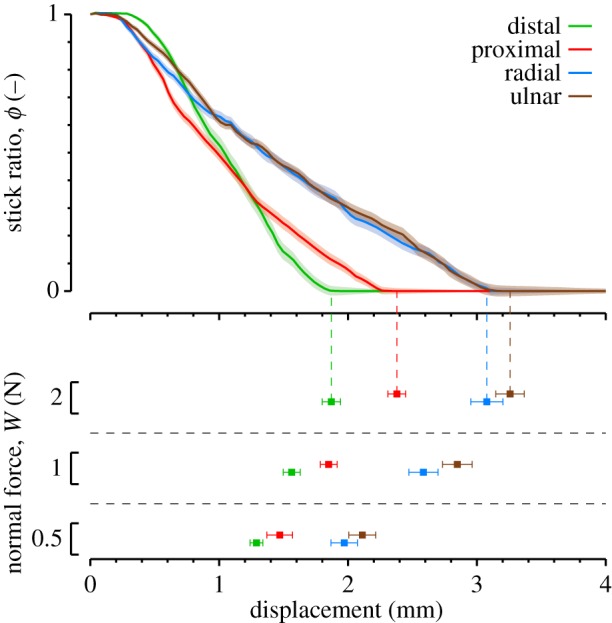
Skin security margin before slippage. Top panel: Evolution of stick ratio as a function of stimulus displacement, for a normal force level of 2 N. Bottom panel: Corresponding displacement observed at the first instant of full slip for different levels of normal force. Colours correspond to direction. Solid traces and squares are means across all trials. Shaded areas and error bars represent the standard errors.

The top panel in figure 9 shows the average evolution of the stick ratio as a function of plate displacement in each direction, for a normal force of 2 N. The lower panel shows the displacement corresponding to the first instant of full slip (i.e. when the *ϕ* = 0). The total plate displacement was larger for the radial and ulnar directions (three-way ANOVA, *p* < 0.001), and was slightly larger for the ulnar than the radial direction (Tukey post hoc test, *p* = 0.03). Higher levels of normal force corresponded to larger displacements (*p* < 0.001). We did not find any significant influence of the displacement speed (*p* = 0.07), even if the full slip seemed to appear sooner for higher speeds.

### 3.4. Steady-state slip

Steady-state slip, defined as the state reached when the displacement field becomes homogeneous (see §2.2.4.), occurred around the same instant when the contact area and tangential force reach a plateau. The average additional displacement between the full and steady-state slip was 2.10 mm (across all trials). Therefore, even after full slip, because of the presence of a displacement gradient, strains took place within the slipping area.

## 4. Discussion

In this study, we analysed the dynamics of the tangential sliding movements of a fingerpad on a smooth glass plate. First, we observed a systematic decrease of the contact area during the initiation of tangential sliding. Based on the Hertz contact, we proposed that this change could be explained by changes in the skin mechanical properties due to the change in the tangential force. Second, the stuck area decreased linearly with the tangential force, with the slope and intercept of this relationship being strongly influenced by the speed and normal force. Third, the stimulus direction highly influenced the shape of the contact and stuck areas. Finally, the skin deformation margin of the fingertip to mechanical stretch varied with the direction of the stimulus and increased with increasing normal force.

### 4.1. Friction

We observed high values and high variability for the CDF. Several studies have been specifically designed to measure friction of the fingerpad on smooth glass [1,29,30]. It was found that, specifically on hydrophilic glass surface, a major factor influencing friction was fingertip moisture. Depending on the initial state of the finger but also on the occlusion time, that is the time the finger keeps contact before sliding, the friction can vary a lot (0.5–4). The individual sweat variation as well as sweat rates can thus explain the variability observed in our data. Despite the stick-to-slip differences observed across directions, no variation of the coefficient of friction across direction was observed, probably due to the small contribution of the skin deformation relative to adhesion in friction.

### 4.2. Variations in the contact area

Under a constant normal force, the contact area was systematically reduced during tangential loading. This reduction was mainly a consequence of the tissues losing contact with the glass plate during tangential traction, rather than a skin strain mechanism within the contact area. To explain this peeling phenomenon, we used the Hertz contact area equation (see equation (2.1)). Several studies have shown [31–33] that this equation accurately predicts the change in contact area with normal force (i.e. through a power law with an exponent less than or equal to 2/3) within a range from 0 to 2 N. Based on the assumption of a Hertzian contact, we hypothesized that the change in the contact area during tangential loading was related to a change in the Young's modulus of the fingerpad. This change in contact area could be described robustly (*R*^2^ around 0.9) by a simple linear relationship between Young's modulus and the tangential force.

Although our hypothesis does not explain the shape of the resulting contact area or the localization of the parts that lose contact, it can explain the contact area reduction in terms of changes in the mechanical properties during shearing. Specifically, contact area reduction is related to the synchronous increase of the tangential force that produces skin stiffening. Such nonlinear stiffening of the fingertip skin has been reported in several works, during normal loading or tangential traction [15,34]. The existence of a threshold traction force to produce a change in the skin properties can be explained by the linear behaviour of the skin for a short amount of traction, ranging between 0.5 and 0.8 N depending on the direction (figure 6*b*). This dual behaviour of the fingertip skin, i.e. soft and elastic under small constraints and much stiffer in the case of higher constraints, has a physical and a functional explanation. The fibrils of the skin collagen fibres network have a randomly coiled structure when relaxed, giving this soft and elastic behaviour under small stresses. But as fibres become oriented and straightened out in the stress direction they start to carry stress and become much stiffer [35]. This skin structure may help to deal with very different tasks ranging from light tactile exploration and precision grip to the handling of heavy loads.

### 4.3. Stuck and slipping parts

As has been previously reported [1,13], we confirmed that above a threshold tangential force, the stick ratio decreased linearly with the tangential force to zero. The presence of this threshold tangential force was previously explained by the existence of an intrinsic value of interfacial shear strength at zero contact pressure [36]. The speed of movement and normal force also had strong influences on the offset and slope parameters (figure 7*b*,*c*), which, in turn, impacted the CDF. Thus, our results are in agreement with Pasumarty *et al*. [30], who showed that the friction coefficient increases with speed within the range of 5–20 mm s^−1^ on smooth glass, and with other authors [37–39], who showed that it decreases with normal force following a negative power law.

### 4.4. Effect of direction

We found important variations in the results depending on the direction. Many factors can explain this phenomenon. For example, the fingertips have a complex geometry (e.g. different layers of the skin superimposed on collagen tissues, the presence of a rigid bone, etc.) that influences the deformation of the finger under normal and tangential loading. Some studies observed complex behaviours during normal and tangential loading, such as a viscoelastic response and stress relaxation [34,40,41]. Under tangential loading, Nakazawa *et al*. [42] measured different stiffness values depending on the direction of the stimulation, with stiffer skin in the proximal–distal (around 1 N mm^−1^) compared to the radial–ulnar direction (around 0.5 N mm^−1^). Similarly, we observed lower skin compliance in the proximal–distal direction (around 2 mm of deformation margin) than in the radial–ulnar direction (around 3 mm of deformation margin), and even found a slightly greater compliance in the ulnar compared to the radial direction. Note that the Earth's gravitational pull generates a tangential load mainly in the ulnar direction during dexterous manipulation.

The angle of attack (i.e. angle between the horizontal glass plate and the distal phalanx of the finger) used in this experiment mimics the typical position adopted by the fingers during gripping and exploration tasks [43], with the whole pad involved in the contact rather than the only tip, used for the rapid manipulation of small objects. This finger position produces a geometrical asymmetry in the proximal–distal direction and might explain the asymmetric measurements observed in these directions. For instance, we observed higher compliance of the skin in the proximal direction relative to the distal case. This geometrical asymmetry also explains previously observed asymmetrical pressure distribution in the contact area, with a distal offset of the centre of pressure [14–16].

The particular pattern of the fingerprint within the contact area might also influence the shape of the stuck area. Wang & Hayward [34] showed that, depending on the direction (along or across the fingerprint ridges), the skin could have different local stiffness values, with high local stiffness along the fingerprint ridges. Our results (figure 8*a*) suggest that, in the contact area, the parts of the finger having fingerprint ridges aligned with the direction of motion had a tendency to slip earlier. Thus, tissues in contact that are locally stiffer seem more likely to slip than softer parts.

### 4.5. Perspectives and limitations

The current results are limited to measurements on the right index finger and in contact with a smooth glass surface. However, it is probable that they would quantitatively extend to the other fingers as well as thumb, and qualitatively extend to other rigid materials with sufficiently low asperities. Further analyses could be done with the current set-up on other materials, such as Plexiglass (hydrophobic). Nevertheless, the video measurements are only possible with transparent material, and many difficulties would arise in the case of non-flat surfaces due to the optical deformation of the image. Many aspects of the contact may differ on other surfaces with different roughness for example. The present work focuses on the contact with a rigid surface, but grip and touch is not limited to soft materials (for example, skin-to-skin contact or grip-enhancing surfaces). Thus, further investigations are needed to extend our measurements, but would need more complex measurement apparatus.

## 5. Conclusion

The mechanics at the point of contact of the fingertip with an object or an explored surface determines the haptic perception. In this work, we show how complex the mechanics can be in a simple sliding event on a flat surface. These mechanisms are important because they play a major role in generating useful tactile information and, consequently, determining the perceptual and motor performances.
